# Supplementary material for: Sub-nanosecond memristor based on ferroelectric tunnel junction
Source: Nat Commun. 2020 Mar 18;11:1439. doi: 10.1038/s41467-020-15249-1 (PMC7080735; doi:10.1038/s41467-020-15249-1)
Supplement: Supplementary file 1 — Supplementary Information [file 41467_2020_15249_MOESM1_ESM.pdf]

Supplementary Information

Sub-nanosecond Memristor Based on Ferroelectric Tunnel Junction

Chao Ma *et al*.

List of contents:

Supplementary Note 1. *I*-*V* loops of the Ag/BTO/NSTO FTJ

Supplementary Note 2. R-V_p hysteresis loops and cyclability at 100 ns and 1.42 ns

Supplementary Note 3. Real-time electrical measurement setup

Supplementary Note 4. Resistive switchings under different pulse voltages of 600 ps

Supplementary Note 5. Endurance and device-to-device variability

Supplementary Note 6. Sub-nanosecond pulse driven synaptic weight modulation

Supplementary Note 7. Transport analyses and band diagrams

Supplementary Note 8. Hall measurements of NSTO substrates

Supplementary Note 9. FTJs with different metal electrodes

Supplementary Note 10. Excluding the occurrence of Ag migration

Supplementary Note 11. High-temperature retention properties

Supplementary Note 12. Artificial neural network (ANN) simulation with FTJs

Supplementary Note 1. ***I-V*** **loops of the Ag/BTO/NSTO FTJ**

The prototype device structure of the Ag/BTO/NSTO (Nb: 0.7 wt%) FTJ is schematically illustrated in the inset of Supplementary Figure 1a. The test voltage was applied to the Ag top electrode, and the NSTO substrate was always grounded through an ohmic contact pad at its backside.

The typical pinched *I-V* hysteresis loops of the FTJ show a memristive characteristic (Supplementary Figure 1a, b), similar to the previous report[1]. In addition, the *I-V* curves reveal a rectifying transport behavior, indicating the existence of the Schottky barrier for MFS-type FTJs.

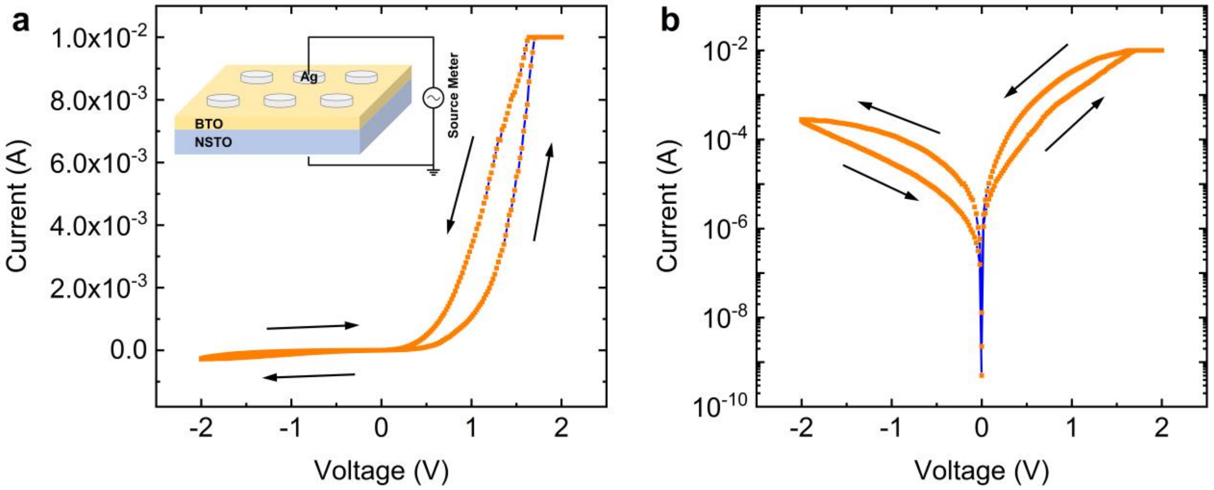

Supplementary Figure 1 *I-V* loops of the Ag/BTO/NSTO FTJ. **a, b** *I-V* curves of a Ag/BTO/NSTO FTJ measured by sweeping the voltage from 0 to 2 V, then 2 V to -2 V, and finally back to 0 V. The arrows indicate the voltage sweeping direction. The inset of **a** is a schematic illustration of the FTJ structure.

Supplementary Note 2. ***R*-V_p hysteresis loops and cyclability at 100 ns and 1.42 ns**

To investigate the memristive behaviors and multi-state switching properties at different operation speeds, the resistance (read at V_r = 0.1 V) *vs.* pulse voltage (V_p) hysteresis loops and cyclability measurements were carried out. Supplementary Figure 2a, b show the voltage pulse sequences for the *R*-V_p and cyclability measurements, respectively. Here, V_r is the reading voltage applied after V_p, and small relative standard deviations (RSD) of reading current fluctuations (< 0.1% for the ON state and < 1% for the OFF state) were observed with V_r in between 0.01 V and 0.1 V.

The *R*-V_p loops measured at 100 ns and 1.42 ns are shown in Supplementary Figure 2c, e. Supplementary Figure 2d, f show the cyclability measurements between the ON state and different

high-resistance states with t_d = 100 ns and 1.42 ns, respectively. The results show that the FTJ has excellent memristive characteristics.

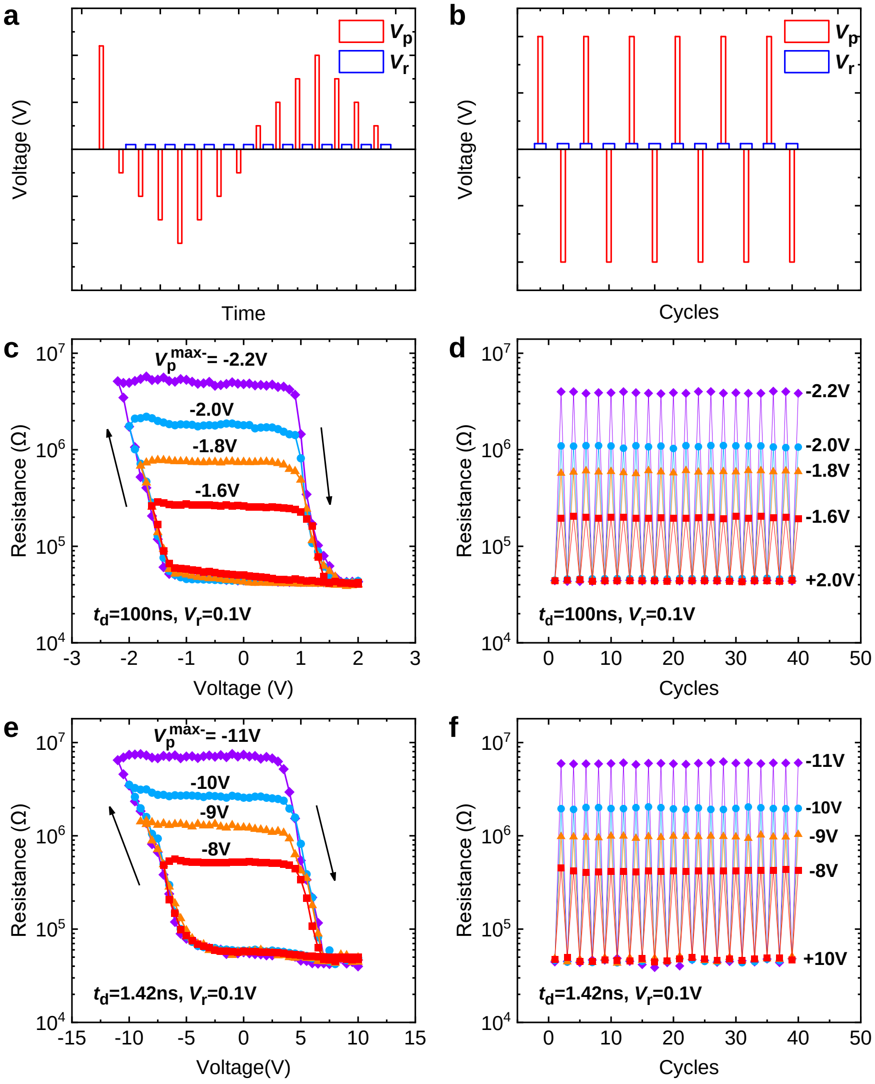

Supplementary Figure 2 R-V_p hysteresis loops and cyclability measurements. **a**, **b** Applied voltage pulse sequences for the R-V_p and cyclability measurements, respectively. R-V_p hysteresis loops and cyclability measurements with pulse durations of **c**, **d** 100 ns and **e**, **f** 1.42 ns. The arrows in **c** and **e** indicate the directions of pulse sequences.

Supplementary Note 3. Real-time electrical measurement setup

To ensure that sub-nanosecond pulses are delivered to the FTJ, we conducted a real-time electrical measurement setup similar to that in the literature[2-5], as shown in Supplementary Figure 3a. A pulse generator (Tektronix PSPL10300B with the shortest pulse duration of about 540 ps) delivers voltage pulses with different amplitudes and durations to induce resistance switchings in

the FTJs. A Keithley 2410 SourceMeter was used to monitor the resistance change of the FTJs after applying write voltage pulses. An oscilloscope (Tektronix DSA70804 with a bandwidth of 8 GHz) was utilized to verify the waveforms applied to the FTJs. A DC/RF switch (Radiall's RAMSES SPDT switch, 0-18 GHz) was used to separate the DC and RF circuit signals. To protect the oscilloscope against overvoltage, -10 dB and -6 dB attenuators were inserted before Channel 1 and Channel 2, respectively.

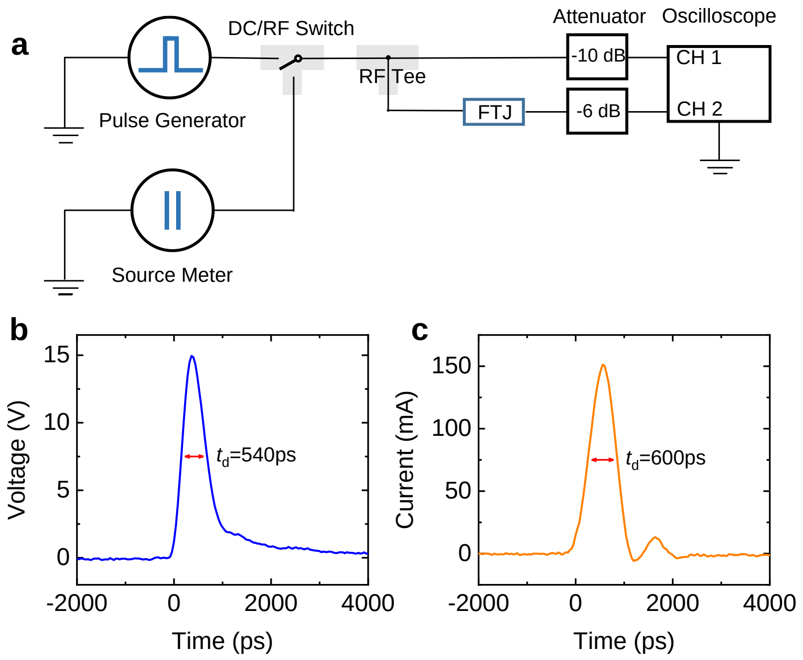

Supplementary Figure 3 Real-time electrical measurement for the Ag/BTO/NSTO FTJ. **a** Schematic description of the real-time electrical measurement setup. **b** Voltage pulse of 540 ps in duration and 15 V in amplitude applied to the FTJ top electrode. **c** Signal transmitted through the FTJ with a duration of 600 ps.

In this way, the voltage pulse applied to the FTJ top electrode can be recorded by using Channel 1 of the oscilloscope. The signal transmitted through the FTJ is also recorded by Channel 2. For example, Supplementary Figure 3b shows that a voltage pulse of 540 ps in duration and 15 V in amplitude was successfully applied to the FTJ. While the signal transmitted through the FTJ shows a pulse duration of about 600 ps, as shown in Supplementary Figure 3c. In other words, the RC delay τ_{RC} extends the 540 ps pulse to 600 ps, and τ_{RC} is estimated to be about (600-540)/2 = 30 ps. Compared with the sub-nanosecond pulse signal, such a small RC delay would not affect the conclusions obviously. In addition, based on Supplementary Figure 3c, the write current density J can be estimated to be about 4×10^3 A cm^{-2}.

Supplementary Note 4. Resistive switchings under different pulse voltages of 600 ps

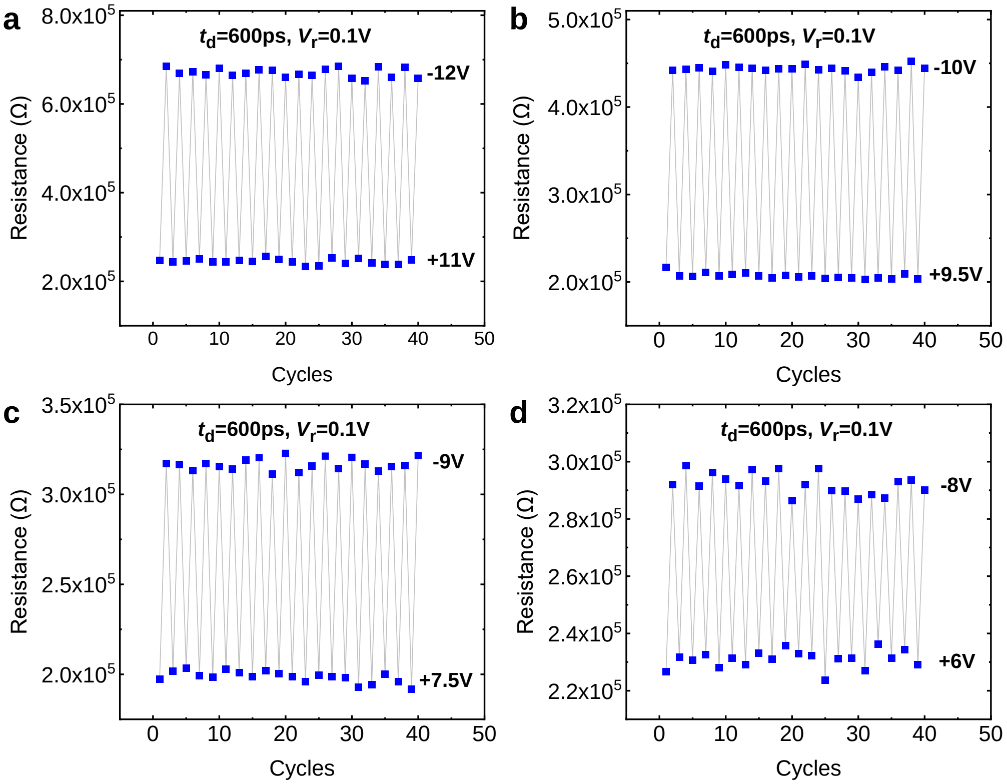

Supplementary Figure 4 Distinguishable resistive switchings by applying 600 ps pulses with different amplitudes.

As demonstrated by the R-V_p loops in Fig. 2c, the resistance of the FTJ changes smoothly between the ON state (~ 4×10^4 Ω) and the OFF state (~ 8×10^6 Ω) with the applications of 600 ps voltage pulses, and there are many intermediate resistive states between ON and OFF states. Although +15 V/-18 V at 600 ps are needed to switch the FTJ between the ON state and the OFF state, respectively, the resistive switchings between the intermediate states require smaller voltages (Supplementary Figure 4). Using +6 V/-8 V (Supplementary Figure 4d), a distinguishable resistive switching between two intermediate states (~ 2.2×10^5 Ω and ~ 3×10^5 Ω) can still be achieved.

Supplementary Note 5. Endurance and device-to-device variability

To test the endurance of the FTJs, a function generator (Agilent 33220A) was utilized to generate square voltage pulses (±3 V amplitude, 100 ns duration, 1 MHz repetition rate) to flip the ferroelectric polarization repeatedly, and the resistance (read at 0.1 V) was monitored after applying voltage pulses of defined numbers. Representative endurance measurement results from two

different FTJs are shown in Supplementary Figure 5a, b. Here, for the first 100 cycles of measurements, the resistances were recorded for each cycle. Then, the representative resistance switching measurements (for 10 cycles) were carried out every 10^n cycles ($n \geq 2$) from 10^n to 10^{n+1} cycles. It can be seen that the FTJs show repeatable resistance switchings up to 10^8-10^9 cycles, one of the best endurance results among the reported FTJs[6-8].

The resistance switching results of 20 different Ag/BTO/NSTO FTJ devices are shown in Supplementary Figure 5c. Despite of the subtle differences among the manually controlled growth conditions for different batches of FTJs as well as the large size (~70 μm in diameter) of the devices, the resistance fluctuations and relative standard deviations of ON (4.08×10^4 Ω – 4.71×10^4 Ω, RSD ~ 4.3%) and OFF (4.80×10^6 Ω – 5.71×10^6 Ω, RSD ~ 5.5%) states are small for the different FTJ samples. The good uniformity in FTJs should be related to the intrinsic ferroelectric nature induced resistance switchings.

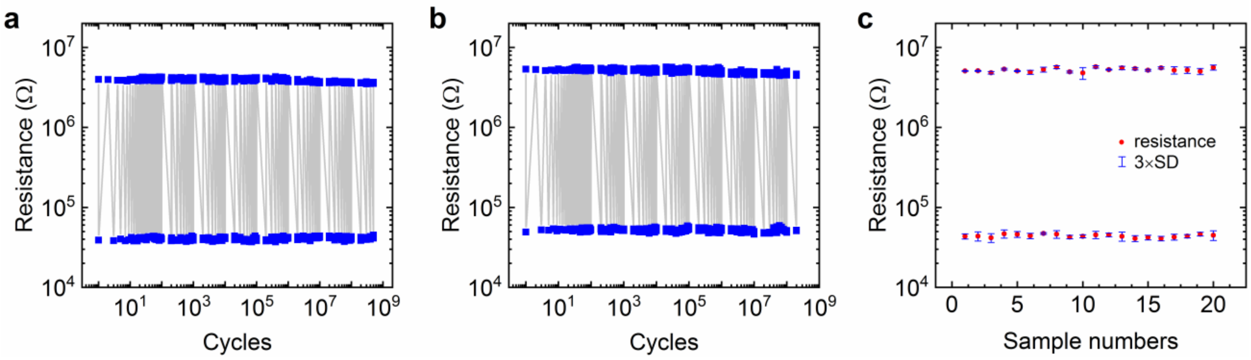

Supplementary Figure 5 Endurance and device-to-device variability measurements. **a, b** Reproducible resistance switchings up to 10^8-10^9 by cycling pulse voltages (±3 V, 100 ns) by using a function generator (Agilent 33220A). **c** Resistances of ON and OFF states for 20 different FTJ devices. The error bar indicates three times the standard deviation (3×SD) of the cycle-to-cycle variations for each FTJ sample.

Supplementary Note 6. Sub-nanosecond pulse driven synaptic weight modulation

To emulate the synaptic weight modification in sub-nanosecond scale, the sub-nanosecond pulse (~600 ps) driven conductance change of the FTJ was measured, as shown in Supplementary Figure 6. The memristor conductance can be manipulated gradually by increasing the amplitude (in a step of 0.5 V) of negative or positive voltage pulses, representing the depression or potentiation of the synaptic weight. In other words, it demonstrates the ability of the FTJs as sub-nanosecond ultrafast synaptic devices.

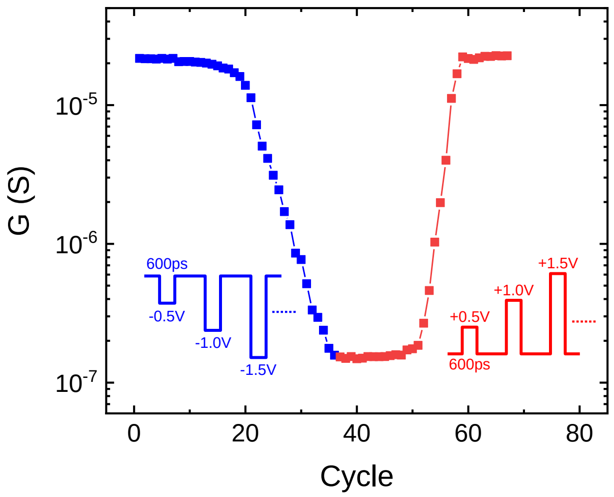

Supplementary Figure 6 Sub-nanosecond pulse driven synaptic weight modulation. The negative (0 to -18 V) and positive pulses (0 to +15 V) with a step of 0.5 V were applied to the FTJ. The pulse duration is ~600 ps, and the device resistance was read with a bias of 0.1 V.

Supplementary Note 7. Transport analyses and band diagrams

The thermally-assisted tunneling current can be described by[9-12]

$$J_F = J_S \exp(qV / E_0) \tag{1}$$

$$J_S = \frac{A^* T^2 \pi^{1/2} E_{00}^{1/2} q^{1/2} [(\Phi_B - V) + \phi_n]^{1/2}}{k_B T \cosh(E_{00}/k_B T)} \times \exp[q(\frac{\phi_n}{k_B T} - \frac{\Phi_B + \phi_n}{E_0})] \tag{2}$$

where $E_0 = nk_B T$, $E_{00} = \frac{qh}{4\pi}\left[\frac{N_D}{m_n^* \varepsilon_r(T)\varepsilon_0}\right]^{1/2}$, and ϕ_n is the difference between the conduction-band minimum and the Fermi level of NSTO. The temperature dependent relative permittivity $\varepsilon_r(T)$ of NSTO can be described by Barrett's formula[11,13]

$$\varepsilon_r(T) = \frac{1635}{\coth(44.1/T) - 0.937} \tag{3}$$

Based on the model, the Φ_B can be extracted from the slope of the linear fitting of $\ln[J_s \cosh(E_{00}/k_B T)/T] - 1/E_0$ on $1/E_0$, as shown in Supplementary Figure 7b, d, f, h and 8b, d, f, h. Furthermore, the linear fitting should be performed in as low voltage bias ranges as possible, typically less than Schottky barrier height, in order to comply with the thermally-assisted tunneling model[10,14]. Besides, the W_d can be estimated from $W_d = (2\varepsilon_0 \varepsilon_r \Phi_B / qN_D)^{1/2}$, where ε_r is ~280 at room temperature according to the Supplementary Eq. 3.

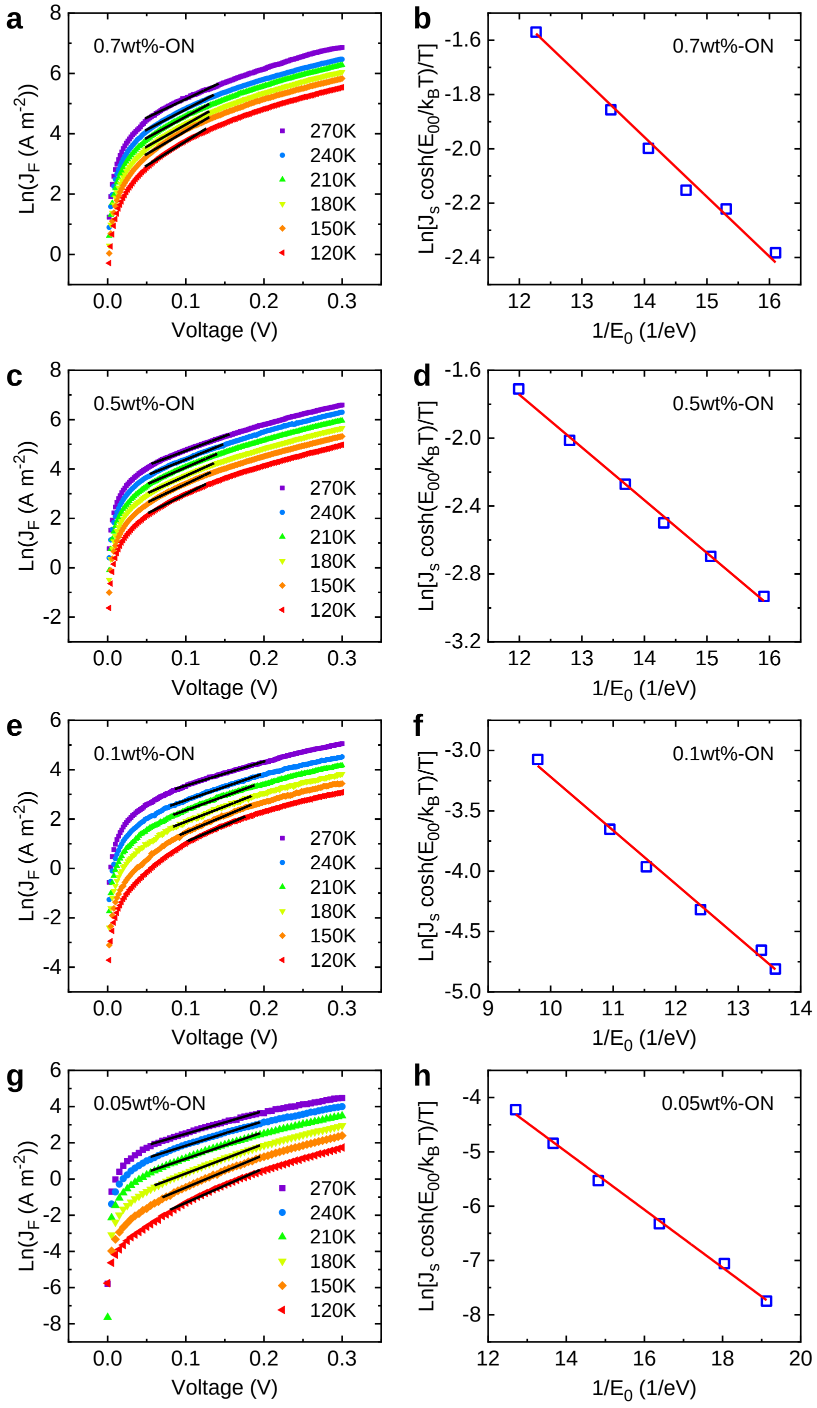

Supplementary Figure 7 Transport analyses for ON states of FTJs with different Nb concentrations. **a**, **c**, **e**, **g** ON states lnJ_F-V curves at various temperatures from 270 to 120 K for the Ag/BTO/NSTO FTJs with Nb concentrations of 0.7, 0.5, 0.1, and 0.05 wt%, respectively. The black solid lines are the fitting results using Supplementary Eq. 1. **b**, **d**, **f**, **h**, ln[$J_s$cosh(E_{00}/$k_B T$)/T] *vs*. 1/E_0 plots for the ON states. The red solid lines are linear fittings using Supplementary Eq. 2.

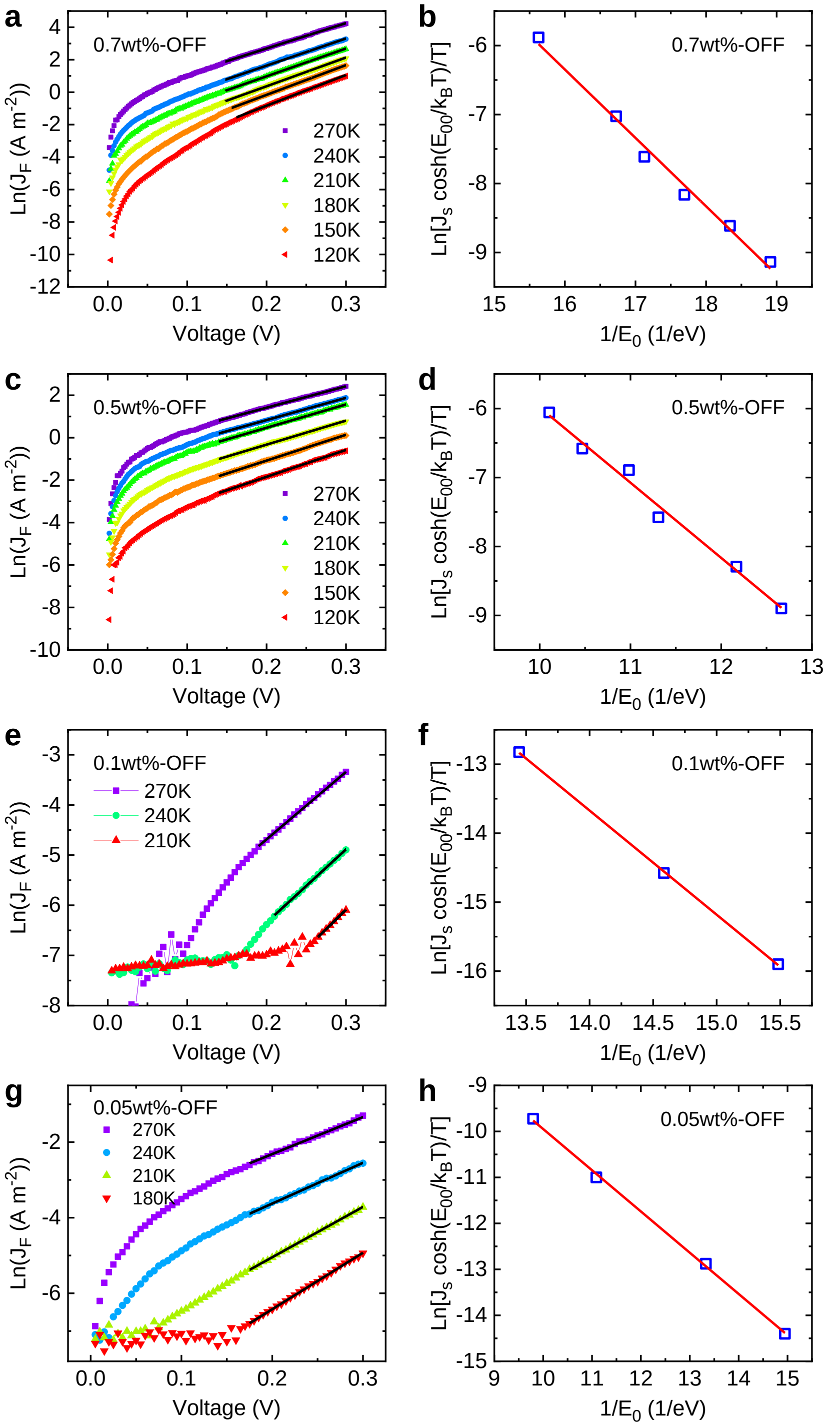

Supplementary Figure 8 Transport analyses for OFF states of FTJs with different Nb concentrations. **a**, **c**, **e**, **g** OFF states lnJ_F-V curves at various temperatures from 270 to 120 K for the Ag/BTO/NSTO FTJs with Nb concentrations of 0.7, 0.5, 0.1 and 0.05 wt%, respectively. The black solid lines are the fitting results using Supplementary Eq. 1. **b**, **d**, **f**, **h**, ln[$J_s$cosh(E_{00}/k_BT)/T] *vs*. 1/E_0 plots for the OFF states. The red solid lines are linear fittings using Supplementary Eq. 2.

According to the fitting results, the schematic diagrams of energy profiles at zero bias for the ON and the OFF states of the FTJs with Nb concentrations of 0.7, 0.5, 0.1, and 0.05 wt% are shown in Supplementary Figure 9. It is obvious that the Schottky barrier height Φ_B and the depleted region width W_d in the OFF state are larger than those in the ON state, indicating that the Schottky barrier can be effectively manipulated by the ferroelectricity[9].

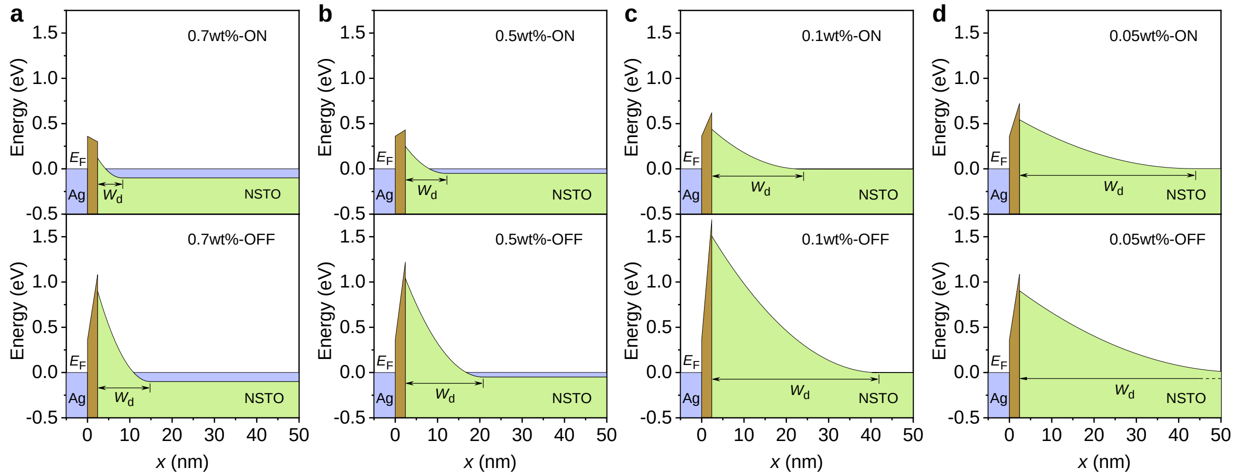

Supplementary Figure 9 Energy profiles of Ag/BTO/NSTO FTJs with various Nb concentrations.

Supplementary Note 8. Hall measurements of NSTO substrates

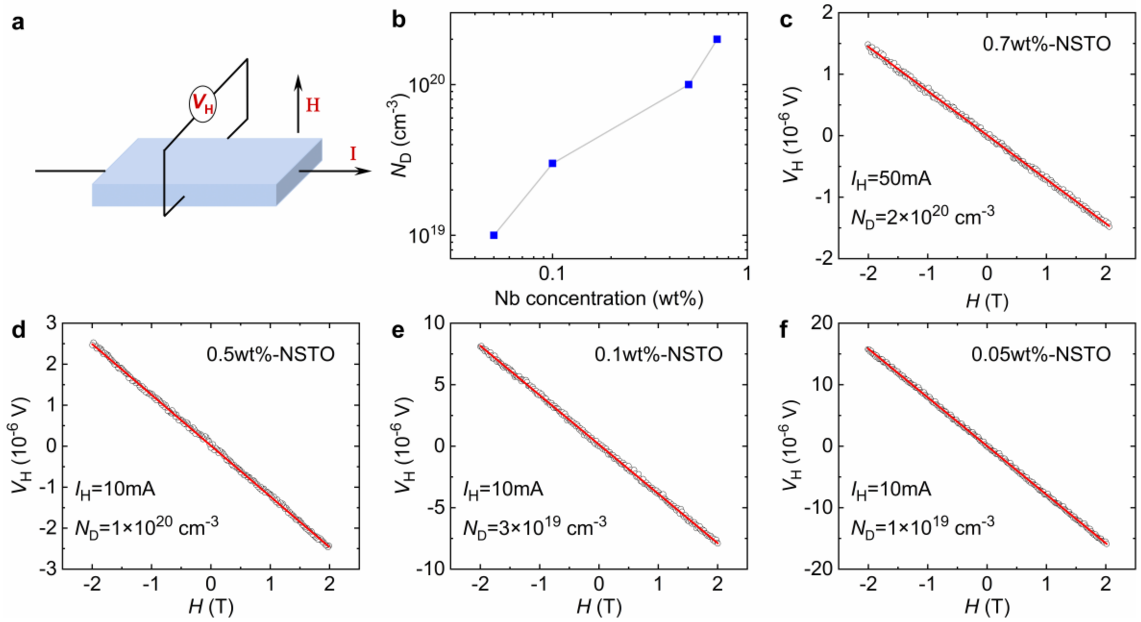

Supplementary Figure 10 Hall measurements of NSTO substrates. **a** Schematic diagram of the Hall measurement. **b** Carrier concentration (N_D) as a function of Nb content. **c-f** Hall voltage V_H as a function of magnetic fields for NSTO substrates with Nb concentrations of 0.7, 0.5, 0.1, and 0.05 wt%, respectively. The solid lines are the linear fitting results.

Supplementary Figure 10 shows the Hall voltage V_H as a function of magnetic fields for NSTO substrates with Nb concentrations of 0.7, 0.5, 0.1, and 0.05 wt%. The carrier concentrations N_D, calculated using $N_D = HI_H / dqV_H$, increase with increasing Nb content, where d ~ 0.5 mm and q = 1.6×10^{-19} C.

Supplementary Note 9. FTJs with different metal electrodes

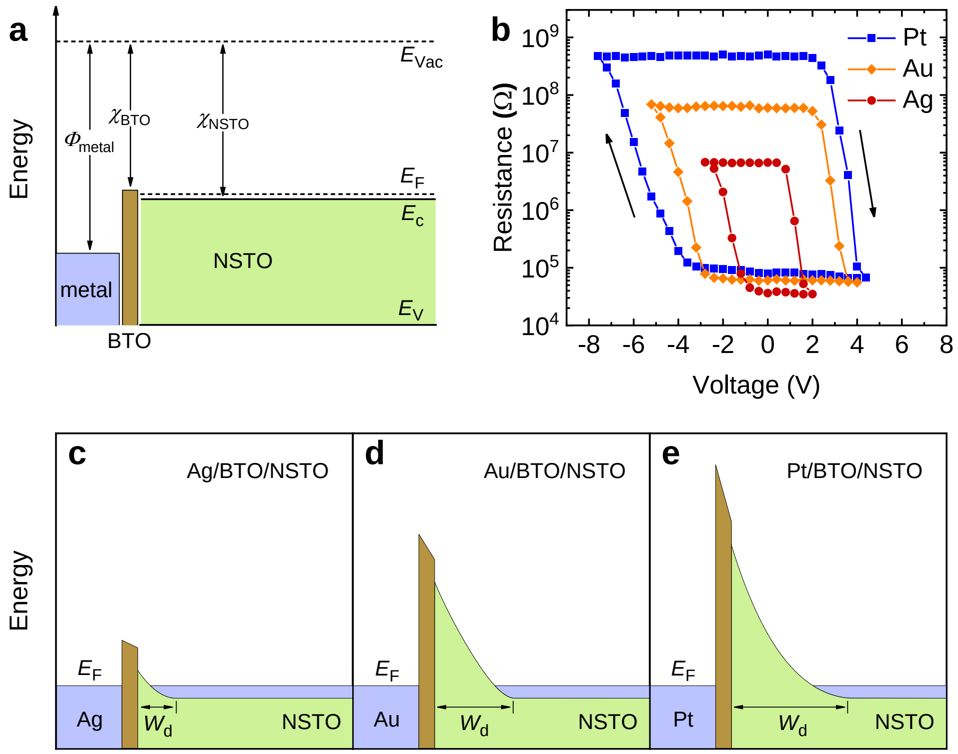

Supplementary Figure 11 FTJs with different metal electrodes. **a** Energy profiles of the separated metal, BTO, and NSTO, where Φ_{metal} is the work function of metal (Ag 4.26 eV, Au 5.1 eV, Pt 5.65 eV), χ_{BTO} = 3.9 eV is the electron affinity of BTO, χ_{NSTO} = 4.0 eV is the electron affinity of NSTO, E_{Vac} is the vacuum level, and E_C, E_V, and E_F are the conduction band minimum, the valence band maximum, and the Fermi level of NSTO, respectively. **b** Resistances measured at 0.1 V *vs.* pulse amplitude V_p with t_d = 100 ns for Ag/BTO/NSTO, Au/BTO/NSTO, and Pt/BTO/NSTO FTJs with a 6 *u.c.* (~2.4 nm) thick BTO barrier and 0.7 wt% Nb concentration. The arrows indicate the direction of pulse sequence. Energy profiles of **c** Ag/BTO/NSTO, **d** Au/BTO/NSTO, and **e** Pt/BTO/NSTO FTJs.

Several metals with different work functions (Ag 4.26 eV, Au 5.1 eV, Pt 5.65 eV) were used as top electrodes for the FTJs to tune the Schottky barrier. According to the semiconductor physics, the

Schottky barrier Φ_B is proportional to the difference between the metal work function Φ_{metal} (the energy difference between the metal Fermi level and the vacuum level) and the electron affinity of the semiconductor χ (the difference between the semiconductor conduction band edge and the vacuum level), namely, $\Phi_B \sim \Phi_{metal} - \chi$, as shown in Supplementary Figure 11a. A smaller work function of the metal electrode will lead to a lower Schottky barrier. Because this Schottky barrier will share a considerable voltage drop from the total applied voltage, lowering the Schottky barrier will increase the partial voltage drop across the BTO barrier. It means that a smaller pulse voltage could flip the ferroelectric domains of BTO in FTJs by using a metal electrode with a smaller work function.

Supplementary Figure 11b shows R-V_p loops (t_d=100 ns) of the MFS-type FTJs with Ag, Au, and Pt metal electrodes. The energy profiles of these FTJs are schematically shown in Supplementary Figure 11c-e. It can be seen that the Pt/BTO/NSTO FTJ shows the biggest current ON/OFF ratio, and this may be one of the reasons why previous researchers mostly used Pt with a high work function as the electrode for their FTJs[9,15]. However, the large Schottky barrier in the Pt/BTO/NSTO FTJ will result in a high operation voltage, which may not be beneficial to practical applications. The utilization of the Ag electrode, by contrast, can greatly reduce the operation voltage, which means that the resistive switching speed will be much faster at a given voltage. Furthermore, it is worth mentioning that the Ag/BTO/NSTO FTJ still presents a current ON/OFF ratio as high as about 2×10^2, which is enough for non-volatile memories with 32 states (Fig. 2).

Supplementary Note 10. Excluding the occurrence of Ag migration

There are some experimental evidences to exclude the occurrence of Ag migration.

1) Figure 1c, d show the HAADF-STEM images from the Ag/BTO/NSTO FTJs at OFF and ON states, respectively. The upward and downward displacements of Ti ions observed at OFF and ON states are consistent with the ferroelectric resistive switchings. It should be noted that there is no Ag migration or Ag filament in BTO, as shown in Fig. 1e, f. This is a direct evidence to exclude the occurrence of Ag migration.

2) The resistance switching characteristics of the FTJs are different from the Ag migration based resistance switchings. The I-V characteristics at ON states for the FTJs are non-linear, following the thermally-assisted tunneling model (Supplementary Figure 7). These are different

from the typically linear *I-V* curves for conduction bridge memories based on Ag filaments at ON states[16].

3) For understanding the resistive switching in the FTJ, we have studied the time-dependent variation of the FTJ resistance and the related ferroelectric domain dynamics behaviors. As shown in Fig. 3, it can be seen that the resistive switching of the Ag/BTO/NSTO FTJ is closely correlated with a nucleation-limited-switching (NLS) model of the ferroelectric domain dynamics[17,18].

4) As shown in Supplementary Figure 11b, not only the FTJ with Ag electrode, but also the FTJs with Au and Pt electrodes show the resistance switching effects. This is also one of the evidences to exclude the occurrence of Ag migration.

All the above experimental results confirm that the resistance switching of the Ag/BTO/NSTO FTJ is caused by ferroelectric polarization switching rather than the conduction bridge based on Ag filaments.

Supplementary Note 11. High-temperature retention properties

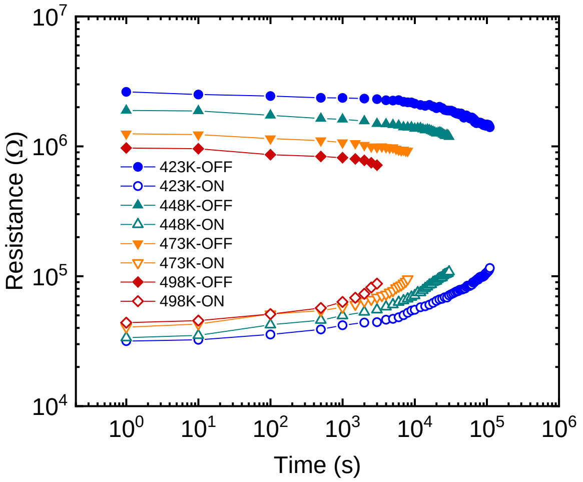

Supplementary Figure 12 High-temperature retention properties. Retention properties of the Ag/BTO/NSTO FTJ at 423, 448, 473 and 498 K.

Supplementary Note 12. Artificial neural network (ANN) simulation with FTJs

ANN Simulation. Similar to earlier reports[19,20], a two-layer perceptron neural network with 784 input neurons, 100 hidden neurons, and 10 output neurons was simulated to implement an online supervised learning on the Modified National Institute of Standard and Technology (MNIST) handwritten digits database, as shown in Supplementary Figure 13. The 784 input neurons

correspond to a 28 × 28 MNIST image, and the 10 output neurons correspond to 10 classes of digits (0 – 9). Generally, for a real memristor crossbar, the inference or classification of a MNIST image could be performed by biasing the top electrode of memristors in the first layer with a set of input voltages (V_{input}, corresponding to small reading voltages without affecting memristor states) whose amplitudes encode an image, then reading the currents from the bottom electrodes of devices in the final layer[19].

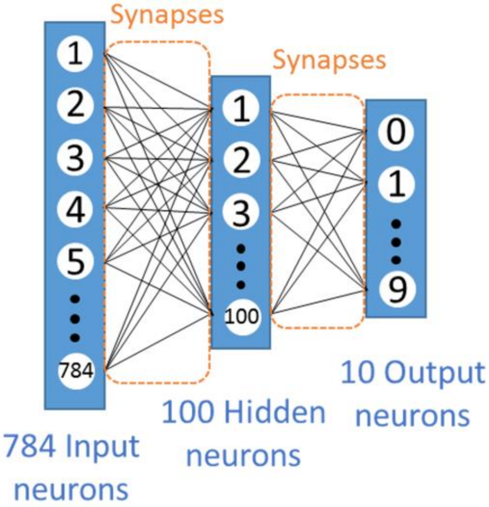

Supplementary Figure 13 Artificial neural network. Schematic diagram of a two-layer neural network.

The training, composed of two stages: feedforward inference and feedback weight update, is based on stochastic gradient descent (SGD) and back propagation algorithms[19]. For each training cycle, 128 images randomly selected from 60000 MNIST digits are set as a batch. The two-layer inference is performed layer by layer sequentially. The input voltage vector to the first layer is a feature vector from the MNIST dataset, and the input vector for the subsequent layer is based on the output vector of the previous layer[19].

Device behavioral model. To implement the SGD algorithm in the memristor crossbar in which the synaptic weight could be positive and negative, the synaptic weight can be encoded as the conductance difference between two paired memristors[21]. Namely, each synapse is implemented with two memristors, so that the total number of memristors in the crossbar is (784×100 + 100×10)×2 = 158800. Typically, the switching of conductance states for each memristor can be achieved by applying voltage pulses, as the conductance (read at 0.05 V) *vs*. pulse number shown in Supplementary Figure 14. Here, the conductance evolutions among 64 conductance states for both

depression (by applying voltage pulses with incremental amplitudes from -0.3 V to -1.8 V with a step of 30 mV, -1.9 V to -3.1 V with a step of 100 mV) and potentiation (by applying voltage pulses from 0.61 V to 0.67 V with a step of 30 mV, 0.7 V to 1.28 V with a step of 20 mV, 1.3 V to 1.59 V with a step of 10 mV) were demonstrated. It can be seen that the conductance depression and potentiation curves are very linear and symmetric. The measurements were repeated by 20 times, and the average conductance *vs*. pulse number can be obtained, as shown in Supplementary Figure 14b. Here, the conductance depression curve was used as the device behavioral model for the simulations, for which the cycle-to-cycle standard deviation relative to the entire conductance range (Δ ~ 2.0%) is small. The cycle-to-cycle and device-to-device variations can be included as random fluctuations following Gaussian distribution in certain ranges.

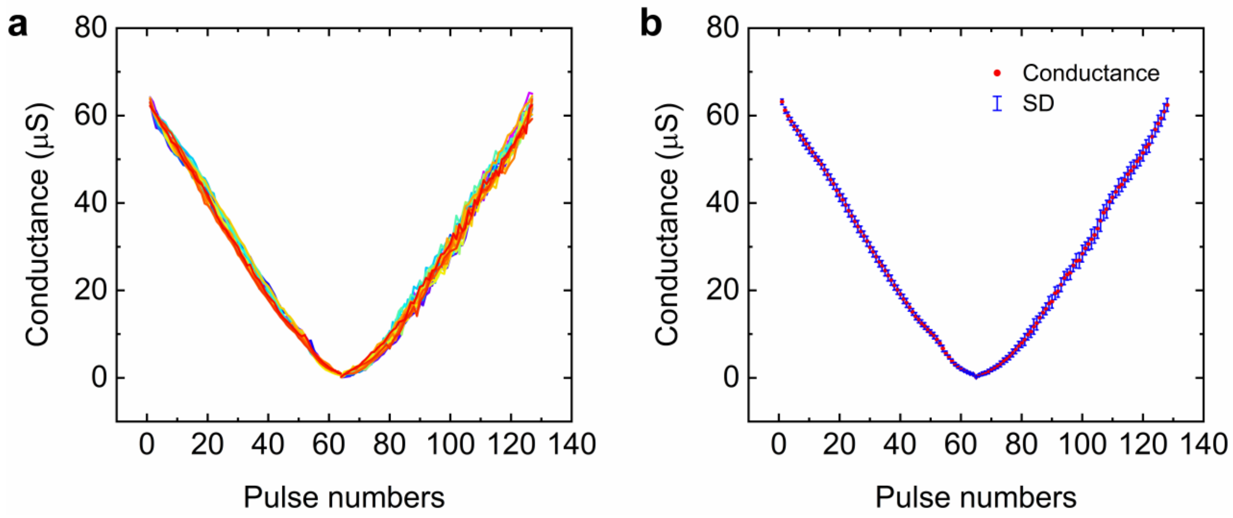

Supplementary Figure 14 Device behavioral model. **a.** Conductance *vs*. pulse number measured for 20 times. **b.** Average conductance *vs*. pulse number. The error bar indicates the standard deviation of the cycle-to-cycle variations for each state.

Supplementary Figure 15a shows the simulation results for the ANN based on the realistic device behavioral model with a cycle-to-cycle variation Δ ~ 2.0%. By comparison, the simulation for an ANN using the ideal synapse (*i.e.*, a memristor which could be tuned to any resistance state without device variations) was also carried out. Similar to the previous report[20], it can be seen that the ANN based on the FTJs (with a device-to-device variation RSD ~ 5%) shows a pattern recognition accuracy > 90%, which is close to the recognition accuracy ~ 97% calculated on the basis of an ideal ANN. It is worth mentioning that the analog computing using memristor arrays can bear certain device variabilities. The effects of the cycle-to-cycle and the device-to-device variations

on recognition accuracies have also been simulated, as shown in Supplementary Figure 15b, c, respectively. It can be seen that the recognition accuracy decreases with increasing cycle-to-cycle and device-to-device variations. Based on the realistic device behavioral model with a cycle-to-cycle variation Δ ~ 2.0% (Supplementary Figure 14), it is still ~87% even supposing the device-to-device variation RSD to be ~35%, consistent with the earlier report[22].

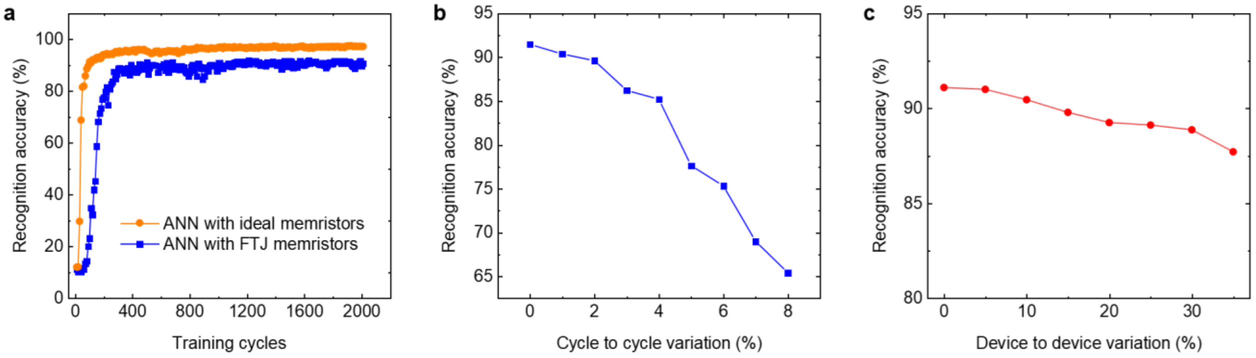

Supplementary Figure 15 Simulated pattern recognition accuracy of the two-layer ANN. **a.** Simulation results based on the FTJ device behavioral model and ideal synaptic devices. **b**. Simulated recognition accuracy *vs*. cycle-to-cycle variation with a fixed device-to-device variation (RSD ~ 5%, see Supplementary Figure 5c). **c.** Simulated recognition accuracy *vs.* device-to-device variation based on the FTJ device behavioral model with a cycle-to-cycle variation Δ ~ 2.0%.

***I-V* nonlinearity**. The forward inference in the computing simulation also depends on the relationship between the input voltage and the output current. In other words, the *I-V* relationship of the memristor will affect the recognition accuracy. Here, to evaluate the effect of realistic *I-V* relationship on computing, the representative *I-V* curves of different resistance states were experimentally characterized, as shown in Supplementary Figure 16a. It can be seen that the nonlinearity decreases with decreasing bias voltage, and it becomes very linear from 0 V to 0.05 V (see Supplementary Figure 16b). Therefore, we could use the realistic *I-V* curves at different V_{input} ranges for ANN simulations to evaluate how the *I-V* nonlinearity affects the computing. With the experimental cycle-to-cycle variation (Δ ~ 2.0%) and the device-to-device variation (RSD ~ 5%), the recognition accuracy *vs*. input voltage range is simulated, as displayed in Supplementary Figure 16c. It can be seen that the recognition accuracy increases with decreasing input voltage range (*i.e.*, with decreasing *I-V* nonlinearity), and it is > 90% below 0.05 V.

For comparison, previous ANN simulations based on realistic performances of various memristors (without considering the effect of *I-V* nonlinearity) can reach a recognition accuracy

~10% – 91% on MNIST digits[20,23]. Thus, one can see that our FTJ could be a good analog synaptic device for neuromorphic hardware systems.

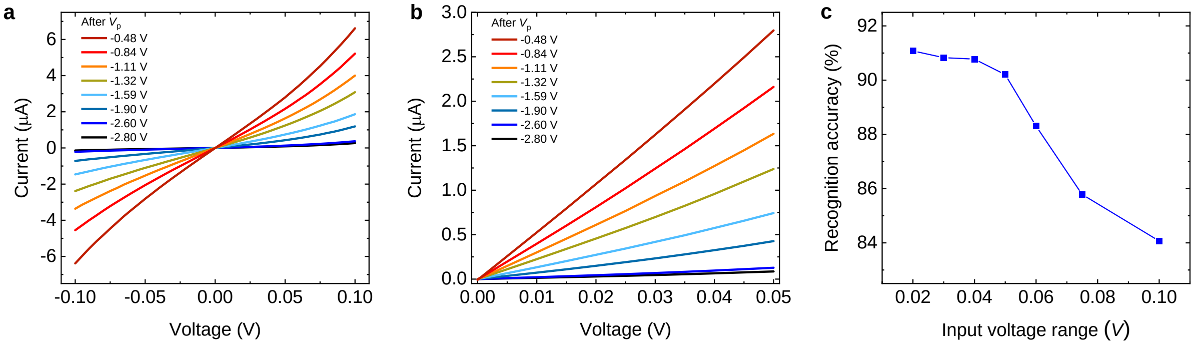

Supplementary Figure 16 *I-V* relationships of the Ag/BTO/NSTO FTJ at different representative resistance states in Supplementary Figure 14 and their effects on simulated recognition accuracy. **a**. *I-V* curves in the range from -0.1 V to 0.1 V. **b**. Enlarged drawing of the *I-V* curves in the range from 0 V to 0.05 V. **c**. Simulated recognition accuracy with different input voltage ranges.

Supplementary References

1. Guo, R., Zhou, Y. X., Wu, L. J., Wang, Z. R., Lim, Z., Yan, X. B., Lin, W. N., Wang, H., Yoong, H. Y., Chen, S. H., Ariando, Venkatesan, T., Wang, J., Chow, G. M., Gruverman, A., Miao, X. S., Zhu, Y. M. & Chen, J. S. Control of synaptic plasticity learning of ferroelectric tunnel memristor by nanoscale interface engineering. *ACS Appl. Mater. Interfaces* **10**, 12862-12869 (2018).
2. Boyn, S., Chanthbouala, A., Girod, S., Carrétéro, C., Barthélémy, A., Bibes, M., Grollier, J., Fusil, S. & Garcia, V. Real-time switching dynamics of ferroelectric tunnel junctions under single-shot voltage pulses. *Appl. Phys. Lett.* **113**, 232902 (2018).
3. Rao, F., Ding, K. Y., Zhou, Y. X., Zheng, Y. H., Xia, M. J., Lv, S. L., Song, Z. T., Feng, S. L., Ronneberger, I., Mazzarello, R., Zhang, W. & Ma, E. Reducing the stochasticity of crystal nucleation to enable subnanosecond memory writing. *Science* **358**, 1423-1427 (2017).
4. Wang, C., Wu, H. Q., Gao, B., Wu, W., Dai, L. J., Li, X. Y. & Qian, H. Ultrafast RESET Analysis of HfO_x-Based RRAM by Sub-Nanosecond Pulses. *Adv. Electron. Mater.* **3**, 1700263 (2017).
5. Havel, V., Fleck, K., Rösgen, B., Rana, V., Menzel, S., Böttger, U. & Waser, R. Ultrafast switching in Ta_2O_5-based resistive memories. *Silicon Nanoelectronics Workshop SNW*, 82-83 (2016).

6. Hu, W. J., Wang, Z. H., Yu, W. L. & Wu, T. Optically controlled electroresistance and electrically controlled photovoltage in ferroelectric tunnel junctions. *Nat. Commun.* **7**, 10808 (2016).
7. Guo, R., Wang, Z., Zeng, S. W., Han, K., Huang, L., Schlom, D. G., Venkatesan, T. & Chen, J. S. Functional ferroelectric tunnel junctions on silicon. *Sci. Rep.* **5**, 12576 (2015).
8. Max, B., Hoffmann, M., Slesazeck, S. & Mikolajick, T. Direct Correlation of Ferroelectric Properties and Memory Characteristics in Ferroelectric Tunnel Junctions. *IEEE J. Electron. Devi.* (2019).
9. Xi, Z. N., Ruan, J. J., Li, C., Zheng, C. Y., Wen, Z., Dai, J. Y., Li, A. D. & Wu, D. Giant tunnelling electroresistance in metal/ferroelectric/semiconductor tunnel junctions by engineering the Schottky barrier. *Nat. Commun.* **8**, 15217 (2017).
10. Cuellar, F. A., Sanchez-Santolino, G., Varela, M., Clement, M., Iborra, E., Sefrioui, Z., Santamaria, J. & Leon, C. Thermally assisted tunneling transport in $La_{0.7}Ca_{0.3}MnO_3/SrTiO_3$:Nb Schottky-like heterojunctions. *Phys. Rev. B* **85**, 245122 (2012).
11. Susaki, T., Kozuka, Y., Tateyama, Y. & Hwang, H. Y. Temperature-dependent polarity reversal in Au/Nb:$SrTiO_3$ Schottky junctions. *Phys. Rev. B* **76**, 155110 (2007).
12. Ruotolo, A., Lam, C. Y., Cheng, W. F., Wong, K. H. & Leung, C. W. High-quality all-oxide Schottky junctions fabricated on heavily doped Nb: $SrTiO_3$ substrates. *Phys. Rev. B* **76**, 075122 (2007).
13. Barrett, J. H. Dielectric constant in perovskite type crystals. *Phys. Rev.* **86**, 118 (1952).
14. Padovani, F. A. & Stratton, R. Field and thermionic-field emission in Schottky barriers. *Solid-State Electron.* **9**, 695-707 (1966).
15. Wen, Z., Li, C., Wu, D., Li, A. D. & Ming, N. B. Ferroelectric-field-effect-enhanced electroresistance in metal/ferroelectric/semiconductor tunnel junctions. *Nat. Mater.* **12**, 617 (2013).
16. Yang, Y. C., Pan, F., Liu, Q., Liu, M. & Zeng, F. Fully room-temperature-fabricated nonvolatile resistive memory for ultrafast and high-density memory application. *Nano Lett.* **9**, 1636-1643 (2009).
17. Boyn, S., Grollier, J., Lecerf, G., Xu, B., Locatelli, N., Fusil, S., Girod, S., Carretero, C., Garcia, K., Xavier, S., Tomas, J., Bellaiche, L., Bibes, M., Barthelemy, A., Saighi, S. & Garcia, V. Learning through ferroelectric domain dynamics in solid-state synapses. *Nat. Commun.* **8**, 14736 (2017).

18. Jo, J. Y., Han, H. S., Yoon, J. G., Song, T. K., Kim, S. H. & Noh, T. W. Domain switching kinetics in disordered ferroelectric thin films. *Phys. Rev. Lett.* **99**, 267602 (2007).
19. Li, C., Belkin, D., Li, Y. N., Yan, P., Hu, M., Ge, N., Jiang, H., Montgomery, E., Lin, P. & Wang, Z. R. Efficient and self-adaptive in-situ learning in multilayer memristor neural networks. *Nat. Commun.* **9**, 2385 (2018).
20. Kim, M.-K. & Lee, J.-S. Ferroelectric Analog Synaptic Transistors. *Nano Lett.* **19**, 2044-2050 (2019).
21. Prezioso, M., Merrikh-Bayat, F., Hoskins, B., Adam, G. C., Likharev, K. K. & Strukov, D. B. Training and operation of an integrated neuromorphic network based on metal-oxide memristors. *Nature* **521**, 61 (2015).
22. Yu, S. M., Chen, P.-Y., Cao, Y., Xia, L. X., Wang, Y. & Wu, H. Q. Scaling-up resistive synaptic arrays for neuro-inspired architecture: Challenges and prospect. *International Electron Devices Meeting (IEDM)*, 17.13.11-17.13.14 (IEEE, Washington, CA, USA, 2015).
23. Jerry, M., Chen, P.-Y., Zhang, J. C., Sharma, P., Ni, K., Yu, S. M. & Datta, S. M. Ferroelectric FET analog synapse for acceleration of deep neural network training. *International Electron Devices Meeting (IEDM)*, 6.2.1-6.2.4 (IEEE, San Francisco, CA, USA, 2017).
